# Supplementary material for: Diversity of Bacterial Biosynthetic Genes in Maritime Antarctica
Source: Microorganisms. 2020 Feb 18;8(2):279. doi: 10.3390/microorganisms8020279 (PMC7074882; doi:10.3390/microorganisms8020279)
Supplement: Supplementary file 1 [file microorganisms-08-00279-s001.pdf]

Supplementary Information

Diversity of Bacterial Biosynthetic Genes in Maritime Antarctica

Adriana Rego [1,2], António G. G. Sousa [1], João P. Santos [1,3], Francisco Pascoal [1], João Canário [4], Pedro N. Leão [1,*] and Catarina Magalhães [1,5,6,*]

[1] Interdisciplinary Centre of Marine and Environmental Research (CIIMAR), University of Porto, 4450-208 Matosinhos, Portugal; adriana.rego@ciimar.up.pt (A.R.); antonio.sousa@ciimar.up.pt (A.G.G.S.); joaofs21@gmail.com (J.P.S.); fpascoal1996@gmail.com (F.P.)
[2] Institute of Biomedical Sciences Abel Salazar (ICBAS), University of Porto, 4050-313 Porto, Portugal
[3] Institute F.-A. Forel, Earth and Environmental Sciences, Faculty of Sciences, University of Geneva, 66, boulevard Carl-Vogt, 1211 Genève 4, Switzerlan
[4] Centro de Química Estrutural at Instituto Superior Técnico, Universidade de Lisboa, Av. Rovisco Pais, 1049-001 Lisboa, Portugal; joao.canario@tecnico.ulisboa.pt
[5] Faculty of Sciences, University of Porto, 4150-179 Porto, Portugal
[6] School of Science, University of Waikato, 3216 Hamilton, New Zealand
*Correspondence: pleao@ciimar.up.pt (P.N.L); cmagalhaes@ciimar.up.pt (C.M.)

Table S1 - Geographic location of the samples included in this study and environmental parameters measured: several trace elements (Cr, Ni, Cu, Zn, As, Pb, Cd and Hg) [1], organic matter (OM) and water content.

Sample	Latitude	Longitude	Cr	Ni	Cu	Zn	As	Pb	Cd	Hg	OM	Water
**AC3**	-62.1671	-58.8517	22.25	18.11	109.37	76.67	18.98	12.86	0.14	0.012	0.1	0.3
**72**	-62.1999	-58.9677	62.23	25.26	71.27	83.13	17.51	15.8	0.15	0.008	2.8	2.3
**87**	-62.1961	-58.9663	65.12	35.61	75.29	76.19	19.59	6.56	0.14	0.012	2.2	2.8
**I1**	-62.1960	-58.9611	68.64	34.35	105.7	216.19	19.36	418.49	1.19	0.074	2.8	0.5
**TR1**	-62.1991	-58.9589	52.23	21	83.19	104.09	18.73	6.66	0.21	0.007	1.8	1.3
**TC3**	-62.1993	-58.9620	63.35	33.49	95.45	79.16	20.5	14.41	0.15	0.025	1.8	1.5
**54**	-62.1994	-58.9656	29.33	16.59	100.31	98.58	17.42	5.19	0.14	0.026	4.1	3.6
**66**	-62.1973	-58.9654	31.41	15.83	65.65	86.97	17.48	7.28	0.61	0.005	2.2	1.8

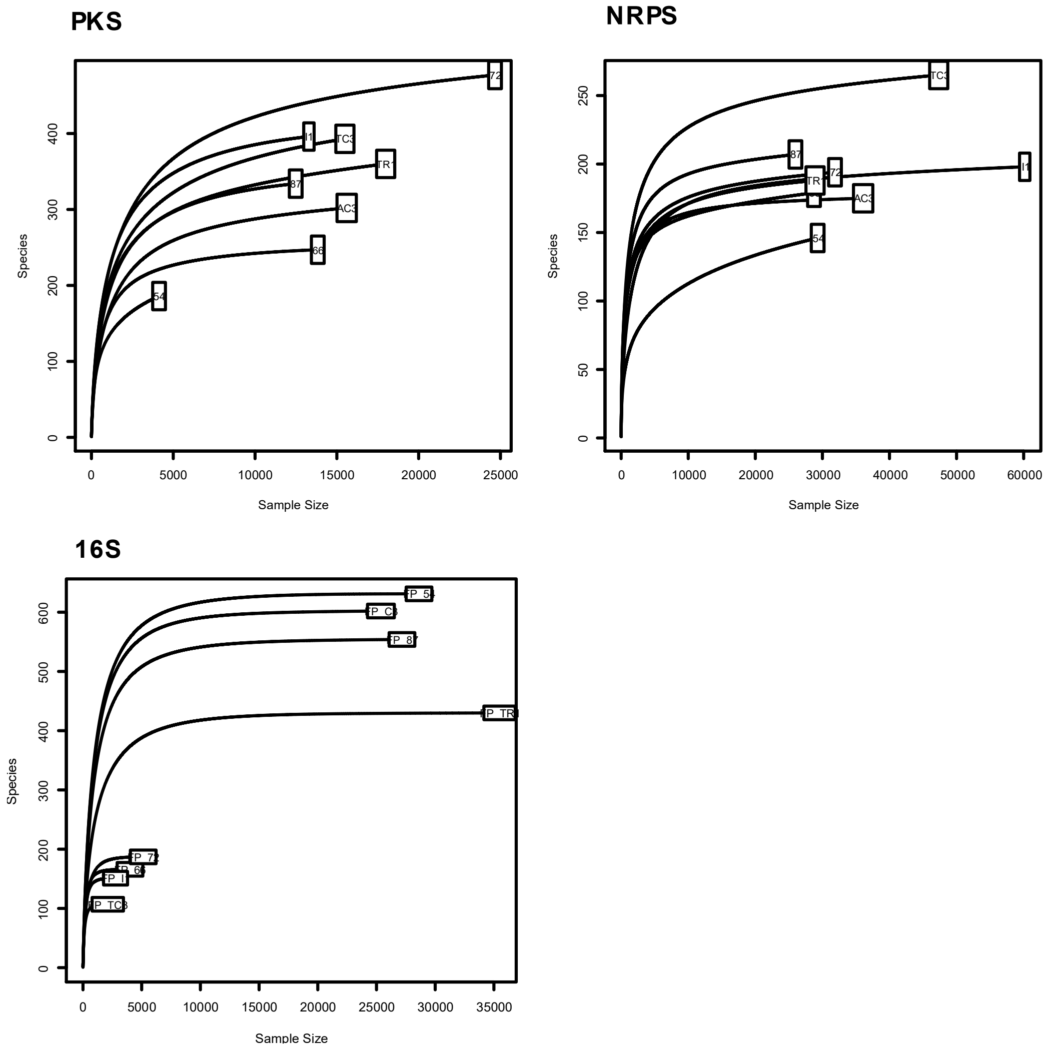

Figure S1 – Rarefaction plot of PKS and NRPS.

Table S2 – Sequence richness and diversity estimates for KS domain of PKS gene. Number of OTUs (grouped at 97% and in a second round at 95%), Chao1, Shannon and Simpson diversity index.

Sample	OTUs	Chao1	se.chao1	Shannon	Simpson
**54**	186	223.1429	15.530469	4.222633	0.9744810
**66**	247	251.4000	3.557533	4.260995	0.9700090
**72**	477	518.7576	15.070831	4.754638	0.9803395
**87**	334	346.6000	6.421924	4.477355	0.9696060
**AC3**	302	322.7143	9.968224	4.059967	0.9520663
**I1**	396	409.0526	6.277387	4.536398	0.9641627
**TC3**	393	436.3636	15.509294	4.654110	0.9789782
**TR1**	360	411.2727	19.724810	4.548058	0.9792373

Table S3– Sequence richness and diversity estimates for AD domain of NRPS gene. Number of OTUs (grouped at 97% and in a second round at 95%), Chao1, Shannon and Simpson diversity index.

Samples	Observed	Chao1	se.chao1	Shannon	Simpson
**54**	146	183.4000	17.144873	2.938287	0.9011069
**66**	179	200.8571	13.883750	3.869712	0.9534230
**72**	194	207.0000	9.258827	3.764392	0.9571343
**87**	207	222.1667	10.859490	3.741089	0.9389775
**AC3**	175	177.0000	2.585619	3.588332	0.9347654
**I1**	198	207.4286	7.257987	2.985950	0.8859579
**TC3**	265	288.3333	13.638560	3.691191	0.9323737
**TR1**	188	197.7500	7.201727	3.682722	0.9389044

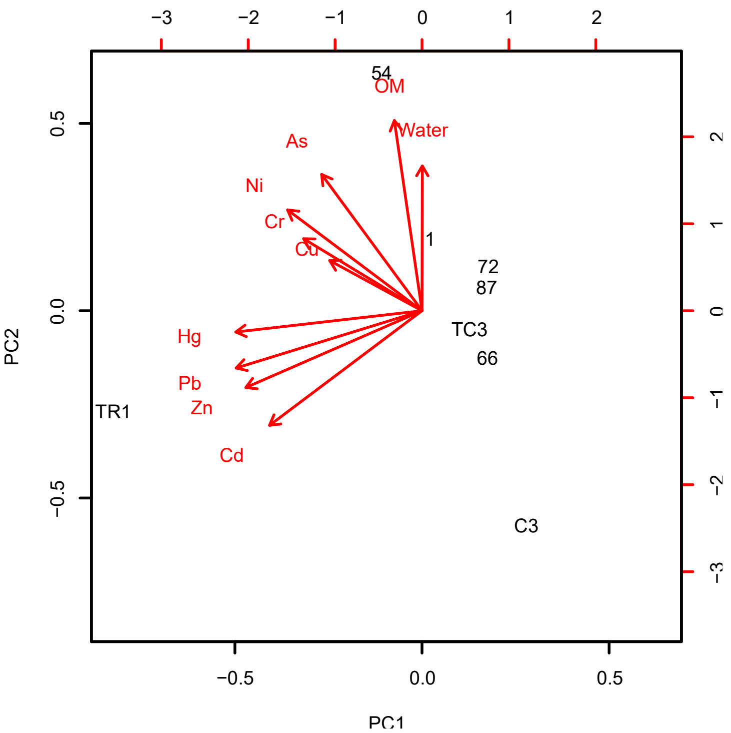

Figure S2 – Principal component analysis (PCA) of the environmental variables measured in this study.

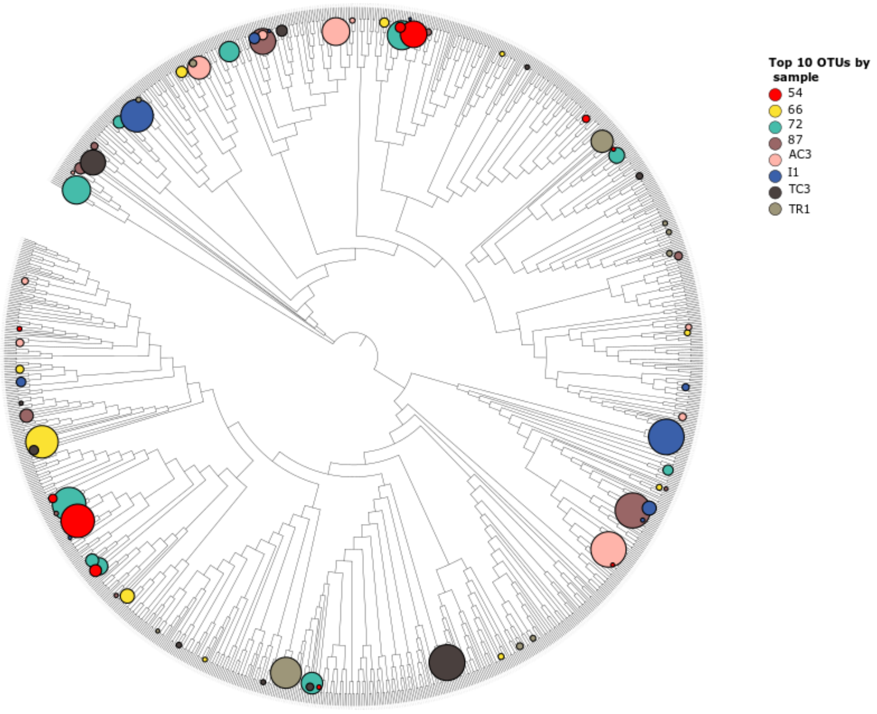

Figure S3 – Maximum likelihood phylogenetic tree of AD domain. The relative abundances of the 10 most abundant OTUs by sample is represented.

Table S4 – Relative abundance of KS domain provenience from actinobacterial genus.

	**Samples**							
	**54**	**66**	**72**	**87**	**AC3**	**I1**	**TC3**	**TR1**
**Actinobacteria**								
Other	3.030303	0.38624952	0.17812611	6.958608	13.2768362	0.0000000	6.97511392	2.60010400
Actinoalloteichus	0.000000	0.00000000	0.00000000	0.000000	0.0000000	64.4444444	0.03505082	0.00000000
Actinocrispum	0.000000	0.00000000	0.00000000	0.000000	0.2824859	0.0000000	0.00000000	0.26001040
Actinoplanes	0.000000	0.00000000	0.00000000	0.000000	0.0000000	0.0000000	3.01437084	0.29901196
Actinosynnema	0.000000	0.00000000	0.00000000	0.119976	0.0000000	0.0000000	0.00000000	0.00000000
Allokutzneria	0.000000	0.00000000	0.03562522	0.000000	2.2598870	0.0000000	0.00000000	0.00000000
Amycolatopsis	0.000000	0.34762457	0.00000000	0.000000	0.0000000	0.0000000	0.00000000	0.00000000
Auritidibacter	0.000000	0.00000000	0.07125045	0.000000	0.0000000	7.5213675	0.00000000	0.00000000
Couchioplanes	0.000000	0.27037466	0.00000000	0.000000	0.0000000	0.0000000	0.00000000	0.00000000
Frankia	0.000000	0.00000000	3.49127182	0.000000	0.0000000	1.5384615	0.00000000	0.00000000
Gordonia	0.000000	0.00000000	0.67687923	2.939412	0.0000000	0.0000000	0.03505082	0.00000000
Herbidospora	0.000000	0.00000000	0.00000000	1.499700	0.0000000	0.0000000	0.00000000	0.00000000
Kibdelosporangium	0.000000	0.00000000	0.00000000	0.000000	2.8248588	0.0000000	0.00000000	0.28601144

Kitasatospora	42.424242	0.00000000	0.00000000	1.019796	0.0000000	0.0000000	0.07010165	0.19500780
Kocuria	0.000000	0.00000000	0.00000000	0.000000	0.0000000	0.8547009	0.00000000	0.00000000
Kribbella	0.000000	0.83043646	0.00000000	0.000000	0.0000000	0.0000000	0.14020329	0.00000000
Lechevalieria	0.000000	1.00424874	0.00000000	0.000000	0.0000000	0.0000000	0.31545741	0.00000000
Micromonospora	6.060606 2	0.64503669	1.78126113	0.299940	14.1242938	6.8376068	3.43498072	0.07800312
Millisia	0.000000	0.00000000	0.00000000	0.000000	0.0000000	0.0000000	3.04942166	0.00000000
Mycobacterium	3.030303 1	3.80842024	57.49910937	6.118776	9.6045198	12.3076923	22.74798458	22.47789912
Mycolicibacter	0.000000	0.00000000	0.99750623	0.000000	0.2824859	0.0000000	0.00000000	0.00000000
Mycolicibacterium	27.272727	0.03862495	0.10687567	0.299940	2.2598870	0.0000000	0.70101647	7.26729069
Nocardia	0.000000	1.58362302	0.00000000	0.239952	0.0000000	0.0000000	13.24921136	12.63650546
Nocardiopsis	0.000000	0.36693704	0.46312789	16.256749	0.0000000	0.0000000	0.00000000	0.18200728
Nonomuraea	0.000000	5.38818076	0.00000000	0.479904	0.0000000	0.0000000	2.06799860	0.00000000
Plantactinospora	0.000000	0.00000000	0.00000000	0.539892	0.0000000	0.0000000	1.78759201	0.00000000
Pseudonocardia	0.000000	7.30011587	0.00000000	2.039592	0.0000000	0.0000000	4.52155626	0.00000000
Rhodococcus	3.030303	0.05793743	0.28500178	22.615477	1.4124294	0.0000000	6.69470733	42.47269891
Saccharothrix	0.000000	0.38624952	0.00000000	0.000000	0.0000000	0.0000000	5.92358920	0.58502340
Salinispora	0.000000 1	8.79103901	7.58817243	0.000000	25.4237288	0.0000000	2.10304942	0.18200728
Streptomyces	15.151515 2	8.79490151	26.71891699	38.572286	28.2485876	5.9829060	23.13354364	9.20436817
Williamsia	0.000000	0.00000000	0.00000000	0.000000	0.0000000	0.0000000	0.00000000	1.23504940
Mycobacterium	0.000000	0.00000000	0.10687567	0.000000	0.0000000	0.5128205	0.00000000	0.03900156

Table S5 – Relative abundance of AD domain provenience from actinobacterial genus.

	**Samples**							
	**54**	**66**	**72**	**87**	**AC3**	**I1**	**TC3**	**TR1**
**Actinobacteria**								
Other	1.190476	2.6940942	2.49140893	2.34220907	36.1400894	5.5103884	12.86186517	1.74208915
Actinoplanes	0.000000	0.0000000	0.02863688	0.73964497	0.0000000	4.2457091	0.00000000	0.91867982
Actinospica	0.000000	3.0656934	0.00000000	0.00000000	0.0000000	0.0000000	0.00000000	0.00000000
Allosalinactinospora	0.000000	0.0000000	0.00000000	0.00000000	0.0000000	2.0776874	0.00000000	0.00000000
Amycolatopsis	0.000000	0.0000000	0.00000000	0.00000000	0.8196721	0.0000000	0.00000000	0.00000000
Arthrobacter	0.000000	0.0000000	0.00000000	0.00000000	2.5335320	0.0000000	0.00000000	0.00000000
Dactylosporangium	0.000000	0.0000000	0.00000000	2.44082840	0.0000000	0.0000000	0.00000000	0.00000000
Frankia	0.000000	4.0743198	0.00000000	1.28205128	0.0000000	0.0000000	0.05008514	0.00000000
Gordonia	0.000000	0.0000000	0.00000000	4.83234714	0.0000000	0.0000000	1.83311630	4.38924804
Herbidospora	0.000000	0.0000000	0.00000000	3.52564103	0.0000000	0.6323397	0.00000000	0.00000000
Kibdelosporangium	0.000000	2.5348374	0.00000000	0.00000000	0.0000000	0.0000000	0.00000000	1.96665532
Kitasatospora	0.000000	0.0000000	0.00000000	4.19132150	0.0000000	0.0000000	0.00000000	1.21810140
Kribbella	0.000000	0.0000000	0.00000000	0.88757396	0.0000000	0.0000000	0.00000000	0.00000000
Lentzea	34.523810	0.0000000	0.00000000	0.41913215	0.0000000	1.5356820	0.00000000	0.00000000
Micromonospora	0.000000	0.0000000	0.00000000	0.00000000	3.2414307	33.4236676	0.00000000	0.00000000
Murinocardiopsis	0.000000	0.0000000	0.77319588	0.00000000	0.0000000	0.0000000	0.00000000	0.00000000
Mycobacterium	26.190476	10.1393497	0.00000000	58.45660750	0.0000000	0.0000000	5.12871882	0.03402518
Nocardia	0.000000	0.5043132	60.05154639	0.39447732	0.0000000	0.0000000	30.97265351	37.39367132
Nonomuraea	0.000000	0.0000000	0.08591065	1.28205128	1.9001490	1.1743451	0.00000000	0.00000000
Rhodococcus	0.000000	8.1884539	3.89461627	1.62721893	29.6199702	6.7750678	22.54833216	28.09799251
Salinispora	0.000000	0.0000000	0.00000000	0.00000000	0.0000000	0.0000000	1.24211159	0.00000000
Sciscionella	0.000000	0.0000000	0.22909507	0.00000000	0.0000000	0.0000000	0.00000000	0.00000000
Smaragdicoccus	0.000000	0.0000000	2.09049255	0.00000000	0.0000000	0.0000000	0.05008514	23.43654304

Streptacidiphilus	0.000000	0.0000000	0.00000000	0.00000000	0.0000000	0.0000000	0.29049384	0.00000000
Streptomyces	38.095238	68.7989383	30.35509737	15.65581854	25.7451565	44.6251129	25.01252129	0.68730861
Streptosporangium	0.000000	0.0000000	0.00000000	0.32051282	0.0000000	0.0000000	0.00000000	0.00000000
Thermostaphylospora	0.000000	0.0000000	0.00000000	1.57790927	0.0000000	0.0000000	0.00000000	0.00000000
Williamsia	0.000000	0.0000000	0.00000000	0.02465483	0.0000000	0.0000000	0.01001703	0.11568561

Table S6 – Relative abundance of KS domain provenience from cyanobacterial genus.

	**Samples**							
	**54**	**66**	**72**	**87**	**AC3**	**I1**	**TC3**	**TR1**
**Cyanobacteria**								
Other	0.0000000	1.3024602	1.07863605	0.0000000	1.0455564	2.56678889	0.00000000	0.2751032
Anabaena	0.0000000	2.6049204	0.06958942	0.0000000	0.0000000	0.00000000	0.47892720	0.0000000
Aphanothece	0.0000000	0.0000000	0.00000000	0.0000000	0.0000000	1.10005238	0.00000000	0.0000000
Calothrix	0.0000000	0.0000000	0.55671538	0.0000000	5.0784167	2.09533787	7.18390805	0.1375516
Chroococcopsis	0.0000000	0.0000000	0.00000000	0.0000000	0.0000000	0.00000000	5.65134100	0.0000000
Coleofasciculus	0.0000000	0.0000000	0.34794711	0.0000000	0.0000000	0.00000000	0.00000000	0.0000000
Crocosphaera	0.5703422	0.1447178	2.08768267	0.0000000	0.0000000	0.00000000	1.43678161	0.0000000
Cyanobium	0.0000000	0.0000000	1.73973556	0.0000000	1.0455564	0.89051860	0.00000000	0.0000000
Cyanosarcina	0.0000000	0.0000000	0.00000000	0.1795332	0.0000000	0.00000000	3.63984674	0.0000000
Desertifilum	0.1901141	0.0000000	0.00000000	0.0000000	0.0000000	0.00000000	2.68199234	0.0000000
Fischerella	0.0000000	0.0000000	0.06958942	0.0000000	0.8215086	2.25248821	0.28735632	0.1375516
Fortiea	0.0000000	0.0000000	0.00000000	0.0000000	0.0000000	0.47145102	7.37547893	0.0000000
Geitlerinema	0.0000000	0.0000000	0.00000000	0.0000000	0.0000000	0.20953379	0.00000000	0.0000000
Gloeothece	0.0000000	0.2894356	0.00000000	0.0000000	0.1493652	0.00000000	0.00000000	0.0000000
Halothece cluster	0.0000000	0.0000000	0.00000000	0.0000000	0.0000000	0.62860136	0.00000000	0.0000000
Hassallia	0.0000000	0.0000000	0.00000000	0.0000000	0.0000000	0.41906757	0.00000000	0.0000000
Hydrococcus	0.0000000	0.0000000	0.00000000	0.3590664	0.0000000	0.10476689	0.00000000	0.0000000
Kamptonema	0.0000000	0.0000000	0.20876827	0.0000000	0.0000000	0.00000000	0.00000000	0.0000000
Leptolyngbya	0.0000000	0.1447178	7.20250522	10.2333932	0.0000000	2.72393924	0.19157088	0.0000000
Limnoraphis	0.0000000	0.0000000	5.21920668	0.0000000	0.0000000	0.00000000	0.00000000	0.0000000
Lyngbya	0.0000000	0.0000000	1.14822547	0.0000000	1.2696042	0.00000000	0.00000000	0.0000000
Mastigocoleus	0.0000000	1.4471780	0.00000000	0.0000000	0.0000000	0.00000000	0.19157088	0.0000000
Microcystis	0.0000000	0.8683068	0.00000000	0.0000000	0.0000000	0.00000000	0.00000000	0.0000000
Moorea	0.0000000	2.7496382	7.82881002	2.8725314	70.0522778	53.79779990	6.70498084	2.7510316
Myxosarcina	0.0000000	0.0000000	0.00000000	0.0000000	0.0000000	0.89051860	0.00000000	0.0000000
Nodularia	0.1901141	1.4471780	8.24634656	0.0000000	0.5974608	0.05238345	0.57471264	4.9518569
Nostoc	30.7984791	47.9015919	59.95128740	13.8240575	13.3681852	16.13410162	26.72413793	39.2022008
Nunduva	0.0000000	1.7366136	0.00000000	0.0000000	0.0000000	0.00000000	0.00000000	0.0000000
Phormidesmis	0.0000000	0.0000000	0.00000000	0.7181329	0.0000000	0.00000000	0.09578544	0.0000000
Planktothrix	0.0000000	4.9204052	0.00000000	0.7181329	0.2240478	2.30487166	0.00000000	0.0000000
Plectonema	0.0000000	1.4471780	0.06958942	0.0000000	0.0000000	0.00000000	0.00000000	8.9408528
Pleurocapsa	0.0000000	0.5788712	0.00000000	0.0000000	0.0000000	0.26191723	0.19157088	5.0894085
Pseudanabaena	0.0000000	0.0000000	0.10438413	0.0000000	3.4353996	0.00000000	0.00000000	0.0000000
Rivularia	0.0000000	0.0000000	1.28740431	0.0000000	0.0000000	0.00000000	0.00000000	0.0000000
Scytonema	68.2509506	20.6946454	0.55671538	63.9138241	2.9126214	10.68622315	33.90804598	0.1375516
Stanieria	0.0000000	4.3415340	0.00000000	0.0000000	0.0000000	0.00000000	0.00000000	0.0000000
Symploca	0.0000000	0.0000000	0.41753653	0.0000000	0.0000000	0.10476689	1.53256705	0.0000000
Tolypothrix	0.0000000	0.0000000	0.00000000	0.7181329	0.0000000	0.00000000	0.38314176	0.0000000

Trichodesmium	0.0000000	0.1447178	0.00000000	0.0000000	0.0000000	0.00000000	0.76628352	0.0000000
Trichormus	0.0000000	6.2228654	1.70494085	6.4631957	0.0000000	2.30487166	0.00000000	38.3768913
Westiellopsis	0.0000000	1.0130246	0.00000000	0.0000000	0.0000000	0.00000000	0.00000000	0.0000000
Moorea	0.0000000	0.0000000	0.10438413	0.0000000	0.0000000	0.00000000	0.00000000	0.00000000

Table S7 – Relative abundance of AD domain provenience from cyanobacterial genus.

	Samples							
	54	66	72	87	AC3	I1	TC3	TR1
**Cyanobacteria**								
Other	57.094477249	12.30684327	0.4418262	5.48621190	0.5956433	31.60124259 1	1.42032571	0.2022927
Acaryochloris	0.000000000	0.00000000	0.0000000	0.05805515	0.0000000	0.00000000	0.55658627	0.0000000
Anabaena	0.000000000	0.00000000	0.0000000	3.30914369	1.0551396	0.00000000	0.00000000	0.0000000
Aphanothece	0.703369225	0.00000000	0.0000000	0.00000000	0.0000000	0.00000000	0.00000000	0.0000000
Calothrix	0.078152136	5.84988962	0.9327442	1.04499274	3.4547311	6.60830274	0.32982890	0.0000000
Chamaesiphon	0.000000000	0.00000000	3.1909671	0.00000000	0.2212389	29.14430952	0.43290043	1.2137559
Chlorogloeopsis	0.000000000	0.00000000	2.0127639	0.00000000	0.0000000	0.00000000	0.00000000	0.0000000
Coleofasciculus	0.000000000	0.55187638	0.0000000	0.14513788	0.0000000	0.00000000	0.00000000	0.0000000
Crinalium	0.000000000	0.00000000	0.0000000	7.16981132	0.0000000	0.00000000	0.00000000	0.0000000
Crocosphaera	0.000000000	5.24282561	0.0000000	0.00000000	0.0000000	0.00000000	0.00000000	0.0000000
Cyanobacterium	0.000000000	0.00000000	0.0000000	0.00000000	1.1402314	0.02824061	0.00000000	0.2697235
Cyanothece	4.307051059	15.50772627	0.0000000	3.16400581	0.0000000	0.00000000	8.49309421	0.0000000
Cylindrospermum	5.748523793	0.00000000	0.0000000	8.85341074	0.3914227	0.00000000 4	3.94970109	0.0000000
Fischerella	0.000000000	0.00000000	0.0000000	0.08708273	0.0000000	0.00000000	0.00000000	0.2697235
Gloeocapsa	0.017367141	0.00000000	0.0000000	0.02902758	0.0000000	1.49675233	1.03071532	6.2710722
Hassallia	0.000000000	0.00000000	0.0000000	0.00000000	0.3914227	0.00000000	0.00000000	0.0000000
Leptolyngbya	0.000000000	0.00000000	0.4909180	0.00000000	0.0000000	0.00000000	0.37105751	3.7761295
Mastigocoleus	0.008683571	48.67549669	0.0000000	0.00000000	0.0000000	0.00000000	4.76190476	0.0000000
Merismopedia	0.000000000	1.10375276	0.0000000	25.51523948	0.2382573	0.00000000 1	1.42032571	0.0000000
Microcoleus	0.000000000	0.00000000	0.0000000	1.07402032	0.0000000	0.00000000	0.26798598	0.0000000
Microcystis	0.000000000	0.00000000	10.9474718	0.00000000	0.8168822	3.78424174	0.00000000	0.0000000
Moorea	0.069468565	0.05518764	0.1472754	2.00290276	8.1688223	0.76249647	2.28818800	0.0000000
Myxosarcina	0.000000000	0.00000000	0.5891016	0.00000000	0.0000000	0.00000000	0.00000000	0.0000000
Nostoc	14.102118791	3.91832230	23.4658812	37.41654572	14.3635126	21.74526970	7.02947846	4.6527310
Oscillatoria	0.000000000	0.00000000	0.0000000	0.00000000	0.6126617	3.75600113	0.00000000	0.0000000
Phormidium	0.000000000	0.00000000	4.0255277	0.00000000	65.7420014	0.11296244	0.22675737 8	3.3445718
Planktothrix	17.749218479	0.00000000	0.4418262	0.00000000	0.0000000	0.28240610	0.02061431	0.0000000
Pseudanabaena	0.008683571	0.00000000	1.0800196	0.31930334	2.3144997	0.36712793	0.00000000	0.0000000
Scytonema	0.104202848	5.68432671	31.7623957	4.15094340	0.3744044	0.00000000	1.93774479	0.0000000
Stanieria	0.008683571	0.00000000	0.0000000	0.17416546	0.0000000	0.00000000	5.46279118	0.0000000
Synechococcus	0.000000000	1.10375276	0.0000000	0.00000000	0.0000000	0.00000000	0.00000000	0.0000000
Tolypothrix	0.000000000	0.00000000	20.4712813	0.00000000	0.0000000	0.31064671	0.00000000	0.0000000
Trichormus	0.000000000	0.00000000	0.0000000	0.00000000	0.1191287	0.00000000	0.00000000	0.0000000

Table S8 – Relative abundance of KS domain provenience from Firmicutes genera.

	Samples							
	**54**	**66**	**72**	**87**	**AC3**	**I1**	**TC3**	**TR1**
**Firmicutes**								
Other	0.000000	1.840491	0.0000000	0.000000	0.000000	0.000000	0.000000	0.000000
Bacillus	0.000000	6.441718	5.3927813	10.294118	4.545455	46.391753	7.118644	2.857143
Brevibacillus	35.057471	8.588957	17.7070064	0.000000	0.000000	0.000000	0.000000	0.000000

Clostridium	0.000000	0.000000	0.0000000	11.029412	0.000000	0.000000	0.000000	0.000000
Enterococcus	1.149425	0.000000	0.3397028	5.514706	15.909091	5.154639	0.000000	2.857143
Lihuaxuella	0.000000	0.000000	0.0000000	0.000000	47.727273	0.000000	0.000000	0.000000
Lysinibacillus	0.000000	0.000000	1.3588110	0.000000	0.000000	0.000000	0.000000	0.000000
Marininema	0.000000	0.000000	0.2972399	0.000000	0.000000	0.000000	0.000000	0.000000
Paenibacillus	63.793103	18.711656	74.9044586	54.044118	31.818182	48.453608	85.649718	94.285714
Ruminiclostridium	0.000000	64.417178	0.0000000	7.352941	0.000000	0.000000	7.231638	0.000000
Sporomusa	0.000000	0.000000	0.0000000	6.250000	0.000000	0.000000	0.000000	0.000000
Thermoflavimicrobium	0.000000	0.000000	0.0000000	5.514706	0.000000	0.000000	0.000000	0.000000

Table S9 – Relative abundance of AD domain provenience from Firmicutes genera**.**

	**Samples**							
	**54**	**66**	**72**	**87**	**AC3**	**I1**	**TC3**	**TR1**
**Firmicutes**								
	0.000000	3.459821	0.0000000	0.0000000	0.000000	0.61309679	0.0000000	0.0000000
**Acetonema**	0.000000	0.000000	0.0000000	1.2562814	0.000000	0.00000000	0.0000000	0.0000000
**Anaerobacterium**	0.000000	0.000000	0.0000000	0.0000000	0.000000	0.00000000	2.0238095	0.4950495
**Aneurinibacillus group**	5.797511	0.000000	0.9018759	0.0000000	15.961801	47.15627446	0.0000000	2.4752475
**Bacillus**	4.609729	19.084821	55.5194805	15.0753769	21.521146	45.38220715	2.9761905	7.5907591
**Brevibacillus**	3.563348	39.174107	26.4550265	26.3819095	2.216917	0.07826768	36.5476190	0.0000000
**Desmospora**	0.000000	0.000000	0.0962001	0.0000000	0.000000	0.02608923	0.0000000	0.1650165
**Desulfocucumis**	0.000000	27.790179	0.0000000	0.0000000	0.000000	0.00000000	0.0000000	0.0000000
**Dorea**	0.000000	0.000000	0.0000000	0.0000000	0.000000	0.00000000	0.0000000	11.0561056
**Fontibacillus**	0.000000	0.000000	0.0000000	0.0000000	0.000000	0.00000000	1.7857143	0.0000000
**Hungateiclostridium**	0.000000	2.120536	0.0000000	0.0000000	0.000000	0.00000000	0.0000000	0.0000000
**Lihuaxuella**	0.000000	0.000000	0.0000000	0.5025126	0.000000	0.00000000	0.0000000	0.0000000
**Oceanobacillus**	0.000000	0.000000	0.0000000	0.0000000	0.000000	0.00000000	2.0238095	0.0000000
**Paenibacillus**	2.743213	4.910714	2.5012025	12.8140704	16.200546	2.16540569	24.4047619	70.2970297
**Paludifilum**	0.000000	0.000000	0.1202501	0.0000000	0.000000	0.00000000	0.0000000	0.0000000
**Ruminiclostridium**	0.000000	0.000000	0.0000000	2.0100503	0.000000	0.00000000	0.2380952	0.0000000
**Seinonella**	0.000000	0.000000	0.0000000	6.1557789	0.000000	0.00000000	0.0000000	0.0000000
**Syntrophomonas**	0.000000	0.000000	0.0000000	0.0000000	0.000000	0.80876598	0.0000000	0.0000000
**Thermoactinomyces**	0.000000	0.000000	0.0000000	0.0000000	0.000000	1.39577355	0.0000000	0.8250825
**Thermoflavimicrobium**	0.000000	0.000000	0.0000000	0.0000000	1.057299	0.00000000	0.0000000	0.0000000
**Tumebacillus**	83.286199	3.459821	14.4059644	35.8040201	43.042292	2.37411949	30.0000000	7.0957096

Table S10 – Relative abundance of KS domain provenience from Verrucomicrobia genera.

	**Samples**							
	**54**	**66**	**72**	**87**	**AC3**	**I1**	**TC3**	**TR1**
**Verrucomicrobia**								
	83.3333333	90.5252317	30.59777	51.7094017	75.59809	41.76072	93.01629531	99.137931
**Chthoniobacter**	15.8730159	8.1359423	28.47011	47.6495726	24.40191	45.82393	5.75324243	0.862069
**Opitutus**	0.7936508	0.2059732	0.00000	0.0000000	0.00000	0.00000	0.43232458	0.000000
**Pedosphaera**	0.0000000	1.1328527	0.00000	0.0000000	0.00000	0.00000	0.00000000	0.000000
**Roseimicrobium**	0.0000000	0.0000000	0.00000	0.2136752	0.00000	0.00000	0.76488194	0.000000
**Verrucomicrobium**	0.0000000	0.0000000	40.93212	0.4273504	0.00000	12.41535	0.03325574	0.000000

Table S11 – Distribution of KS OTUs with higher identity (above 80%) to the MiBIG database.

OTU_ID	BGC_acession	main_product	Identity	Query cover	evalue	Bioactivity
**Uniq540**	BGC0000148	spinosad	90.000	99	1.70e-33	Insecticide[2]
**Uniq1276**	BGC0001597	fluvirucin b2	89.474	99	1.03e-26	Antifungal and antiviral [3]
**Uniq1728**	BGC0001700	niphimycins C-E	88.750	99	4.96e-41	Antibiotic [4]
**Uniq155**	BGC0000021	apoptolidin	88.710	86	3.06e-29	Cytotoxic [5]
**Uniq623**	BGC0000021	apoptolidin	88.710	80	2.20e-29	Cytotoxic [5]
**Uniq4837**	BGC0000994	FK520	87.931	87	5.50e-27	Immunosupressant [6]
**Uniq477**	BGC0001199	akaeolide	87.719	98	4.73e-25	Antibiotic [7]
**Uniq4169**	BGC0001619	ibomycin	87.719	93	1.61e-25	Antifungal [8]
**Uniq3055**	BGC0000105	nanchangmycin	87.500	99	4.16e-39	Antibiotic [9]
**Uniq44**	BGC0000105	nanchangmycin	87.500	99	4.10e-40	Antibiotic [9]
**Uniq1866**	BGC0000954	ajudazol	86.842	84	1.28e-37	Antifungal [10]
**Uniq3083**	BGC0001013	meridamycin	86.420	89	1.77e-39	Nonimmunosuppressive [11]
**Uniq3683**	BGC0000954	ajudazol	86.207	99	1.16e-46	Antifungal [10]
**Uniq29**	BGC0001597	fluvirucin b2	85.965	99	8.80e-26	Antifungal and antiviral [3]
**Uniq66**	BGC0000086	lasalocid	85.965	83	4.27e-26	Antibiotic [12]
**Uniq2030**	BGC0001204	versipelostatin	85.915	93	8.55e-28	Antitumor [13]
**Uniq2920**	BGC0000002	aculeximycin	85.714	76	3.03e-23	Antibiotic [14]
**Uniq4051**	BGC0000105	nanchangmycin	85.714	100	6.83e-41	Antibiotic [9]
**Uniq62**	BGC0001470	macrobrevin	85.366	99	1.10e-42	Antibiotic [15]
**Uniq940**	BGC0000075	hygrocin	85.246	99	1.30e-25	Antitumor [16]
**Uniq368**	BGC0001200	lorneic acid A	85.000	99	1.04e-39	PDE5 inhibition activity[17] [17]
**Uniq5011**	BGC0000050	E-837	84.932	92	4.88e-34	electron transport inhibitor [18]
**Uniq4143**	BGC0001559	crocagin A / crocagin B	84.810	95	1.08e-37	[19]
**Uniq2579**	BGC0001165	curacin	84.615	79	6.44e-37	Antimitotic, Antiproliferative, and Brine Shrimp Toxic [20]
**Uniq2154**	BGC0000086	lasalocid[21]	84.615	93	5.10e-37	Antibiotic [21]
**Uniq3137**	BGC0000179	etnangien	84.483	99	4.46e-27	Antibiotic [22]
**Uniq852**	BGC0001200	lorneic acid A	84.127	81	2.29e-27	PDE5 inhibition activity (Zhou et al. 2015)
**Uniq3161**	BGC0001700	niphimycins C-E	83.871	99	5.74e-43	Antibiotic [4]
**Uniq4274**	BGC0000114	nigericin	83.750	58	2.52e-37	Antibiotic [23]
**Uniq810**	BGC0001559	crocagin A / crocagin B	83.750	98	2.18e-39	[19]
**Uniq2814**	BGC0001338	citrinin	83.750	99	3.00e-40	Micotoxin [24]
**Uniq324**	BGC0001338	citrinin	83.750	99	3.00e-40	Micotoxin [24]
**Uniq2046**	BGC0000122	[25] A	83.750	99	2.49e-38	multidrug resistance reversal agent [25]
**Uniq5107**	BGC0000122	phenylnannolone A	83.750	99	1.19e-39	multidrug resistance reversal agent [25]
**Uniq503**	BGC0001287	chaxamycin A-D	83.750	78	3.27e-37	Antibiotic [26]
**Uniq753**	BGC0000042	cremimycin	83.750	84	5.21e-38	Antibiotic [27]
**Uniq2553**	BGC0001066	kendomycin	83.750	99	6.41e-36	Antibiotic [28]
**Uniq4297**	BGC0001165	curacin	83.636	84	9.57e-26	Antimitotic, Antiproliferative, and Brine Shrimp Toxic [20]
**Uniq3486**	BGC0001619	ibomycin	83.544	94	1.30e-36	Antifungal [8]
**Uniq1208**	BGC0000172	basiliskamides	82.927	99	9.31e-42	Antifungal and antibiotic [29]
**Uniq1207**	BGC0001099	kalimantacin / batumin	82.716	99	5.35e-43	Antibiotic [30]
**Uniq1559**	BGC0001357	carbamidocyclophane A-U	82.667	96	4.33e-36	Cytotoxic and antibiotic [31]
**Uniq265**	BGC0001200	lorneic acid A	82.540	81	6.21e-27	PDE5 inhibition activity [4]
**Uniq4801**	BGC0001520	aurantinins B-D	82.500	99	5.17e-40	Antibiotic [32]
**Uniq4002**	BGC0001125	puwainaphycins	82.500	97	1.01e-38	Cytotoxic [33]
**Uniq1922**	BGC0000122	phenylnannolone A	82.500	99	3.94e-37	multidrug resistance reversal agent [25]
**Uniq1733**	BGC0001357	carbamidocyclophane A-U	82.500	99	2.82e-39	Cytotoxic and antibiotic [31]
**Uniq2850**	BGC0001357	Carbamidocyclophane A-U	82.500	99	1.82e-39	Cytotoxic and antibiotic [31]
**Uniq4564**	BGC0001287	chaxamycin A-D	82.500	78	4.05e-37	Antibiotic [26]
**Uniq655**	BGC0001199	akaeolide	82.500	85	2.52e-37	Antibiotic [7]

**Uniq4940**	BGC0001349	heronamide	82.500	99	6.20e-41	n.d. [34]
**Uniq149**	BGC0001034	pellasoren	82.456	83	1.54e-25	Cytotoxic [35]
**Uniq879**	BGC0000111	neocarzilin	82.456	83	1.40e-25	Cytotoxic [36]
**Uniq3503**	BGC0000184	sorangicin	82.051	98	5.01e-39	Antibiotic [37]
**Uniq924**	BGC0001099	kalimantacin / batumin	81.944	98	6.32e-49	Antibiotic [30]
**Uniq3379**	BGC0000163	tetronasin	81.944	94	2.93e-33	Antibiotic [38]
**Uniq4882**	BGC0000183	paenimacrolidin	81.707	99	7.12e-44	Antibiotic [39]
**Uniq1630**	BGC0001559	crocagin A / crocagin B	81.667	99	2.74e-26	[19]
**Uniq4087**	BGC0001034	pellasoren	81.667	83	1.01e-26	Cytotoxic [35]
**Uniq1978**	BGC0000954	ajudazol	81.481	99	1.46e-28	Antifungal [10]
**Uniq4903**	BGC0001770	sceliphrolactam	81.481	84	2.38e-38	Antifungal [40]
**Uniq3461**	BGC0001066	kendomycin	81.481	92	3.48e-27	Antibiotic [28]
**Uniq1662**	BGC0001737	phenalamide	81.333	96	1.43e-33	HIV-1 inhibitor [41]
**Uniq1810**	BGC0001470	macrobrevin	81.250	99	5.80e-41	Antibiotic [15]
**Uniq2659**	BGC0000954	ajudazol	81.250	100	1.41e-35	Antifungal [10]
**Uniq46**	BGC0000954	ajudazol	81.250	87	1.38e-35	Antifungal [10]
**Uniq922**	BGC0001559	crocagin A / crocagin B	81.250	99	2.53e-36	[19]
**Uniq1066**	BGC0001125	puwainaphycins	81.250	97	3.81e-38	Cytotoxic [33]
**Uniq3016**	BGC0001125	puwainaphycins	81.250	99	8.98e-37	Cytotoxic [33]
**Uniq1724**	BGC0001024	myxothiazol	81.250	98	7.30e-38	Antibiotic [42]
**Uniq3169**	BGC0000954	ajudazol	81.034	100	1.89e-21	Antifungal [10]
**Uniq120**	BGC0001298	4-Z-annimycin	81.013	74	7.79e-36	Inhibition of sporulation in certain Actinobacteria [43]
**Uniq1325**	BGC0001165	curacin	80.723	99	9.43e-40	Antimitotic, Antiproliferative, and Brine Shrimp Toxic [20]
**Uniq1850**	BGC0000027	azaphilone	80.556	94	1.94e-37	antimicrobial, antiviral, cytotoxic, anticancer, and anti-inflammatory [44]
**Uniq135**	BGC0001470	macrobrevin	80.488	99	9.97e-39	Antibiotic [15]
**Uniq757**	BGC0000954	ajudazol	80.488	75	1.58e-35	Antifungal [10]
**Uniq834**	BGC0001491	67-121C	80.488	99	3.01e-35	Antifungal [45]
**Uniq524**	BGC0001099	kalimantacin / batumin	80.488	99	1.98e-37	Antibiotic [30]
**Uniq771**	BGC0000153	stigmatellin	80.488	99	6.57e-36	Antibiotic [46]
**Uniq2192**	BGC0001663	merocyclophane C / merocyclophane D	80.357	99	5.52e-26	Cytotoxic [47]
**Uniq3359**	BGC0000183	paenimacrolidin	80.247	99	1.11e-42	Antibiotic [39]
**Uniq367**	BGC0000183	paenimacrolidin	80.247	99	3.77e-41	Antibiotic [39]

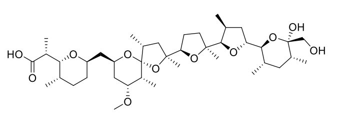

(1)

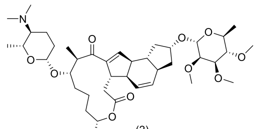

(2)

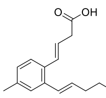

(3)

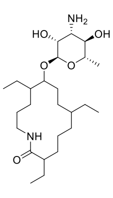

(4)

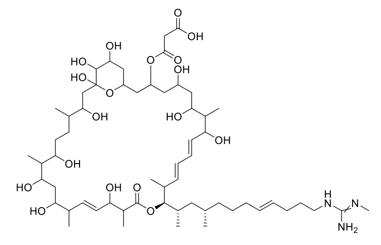

(5)

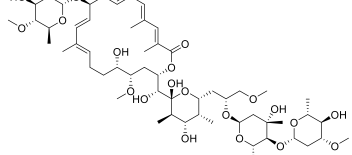

(6)

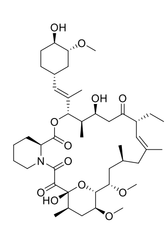

(7)

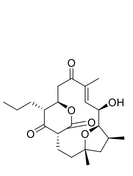

(8)

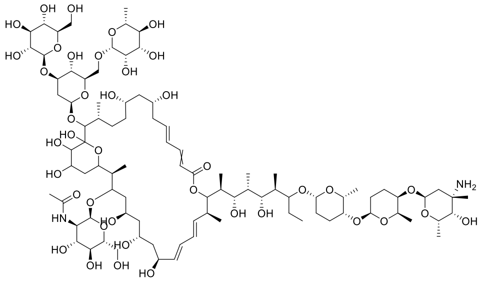

(9)

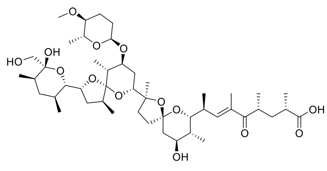

(10)

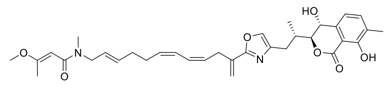

(11)

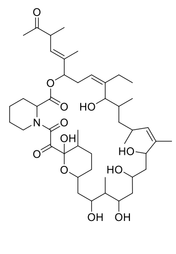

(12)

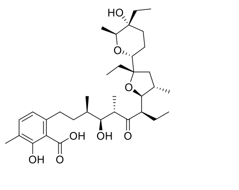

(13)

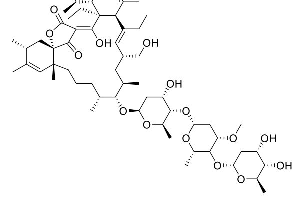

(14)

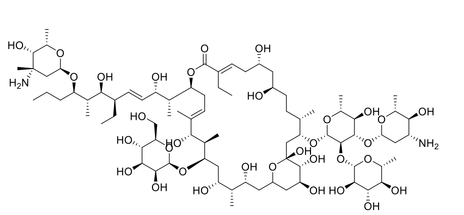

(15)

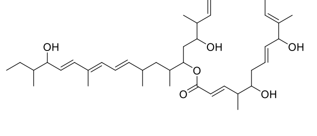

(16)

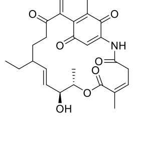

(17)

Figure S4 –For KS domain, 24 OTU sequences were associated with over 85% of identity to BGCs involved in the production of 17 different compounds in MiBiG database. Their chemical structures, presented here, were retrieved using ChemDraw v19.0.1.28. (1) – nigericin, (2) – spinosad, (3) – lorneic acid A, (4) – fluviricin b2, (5) – niphymicin, (6) – apoptolidin, (7) – FK520, (8) – akaeolide, (9) – ibomycin, (10) – nanchangmycin, (11) – ajudazol A, (12) – meridamycin, (13) – lasalocid, (14) – versipelostatin, (15) – aculeximycin, (16) – macrobrevin, (17) - hygrocin A.

Table S12 – Distribution of AD OTUs with higher identity (above 80%) to the MiBIG database.

OTU_ID	BGC_acc.	main_product	Identity %	query cover (%)	E-value	Bioactivity
**Uniq443**	BGC0001519	aurantimycin	95.833	97	1.08e-24	Antibiotic and cytotoxic [48]
**Uniq1538**	BGC0000349	erythrochelin	87.931	91	1.31e-29	Siderophore [49]
**Uniq3185**	BGC0000349	erythrochelin	87.500	91	5.96e-44	Siderophore [49]
**Uniq6572**	BGC0000461	WAP-8294A_2 (lotilibcin)	87.273	97	9.70e-26	Antibiotic [50]
**Uniq7256**	BGC0000413	pyoverdine	86.207	99	7.69e-26	Siderophore [51]
**Uniq482**	BGC0001036	polyoxypeptin	84.810	99	8.39e-37	Apoptotic[52]
**Uniq7256**	BGC0000413	pyoverdine	84.810	99	5.18e-42	Siderophore [51]
**Uniq315**	BGC0001312	viscosin	82.759	95	1.92e-20	Biosurfactant, antibiotic and antiviral [53]
**Uniq1441**	BGC0000331	cyanopeptin	82.759	99	5.75e-32	Protease inhibitor[54]
**Uniq3128**	BGC0000331	cyanopeptin	82.759	99	1.84e-31	Protease inhibitor[54]
**Uniq3997**	BGC0000413	pyoverdine	82.759	99	2.48e-28	Siderophore [51]
**Uniq577**	BGC0001328	rakicidin A / rakicidin B	82.456	99	1.13e-22	Cytotoxic [55]
**Uniq772**	BGC0001657	lysocin	81.481	97	1.89e-25	Antibiotic [56]
**Uniq6572**	BGC0000461	WAP-8294A2 (lotilibcin)	81.132	97	9.40e-21	Antibiotic [50]
**Uniq1939**	BGC0001125	puwainaphycins	81.034	99	2.36e-29	Cytotoxic [33]
**Uniq3781**	BGC0001028	nostopeptolide	81.034	99	1.26e-28	Unknown
**Uniq6075**	BGC0000397	nostocyclopeptide	81.034	99	1.57e-28	Antitoxin [57]
**Uniq7312**	BGC0001608	glidopeptin	80.769	97	1.39e-38	Weak antibiotic and cytotoxic [58]
**Uniq31**	BGC0001657	lysocin	80.702	99	1.44e-25	Antibiotic [56]

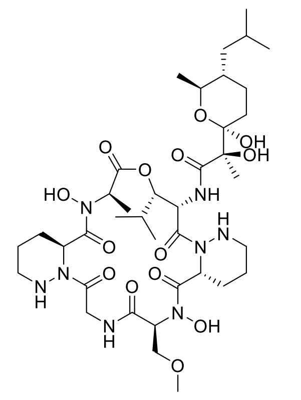

(1)

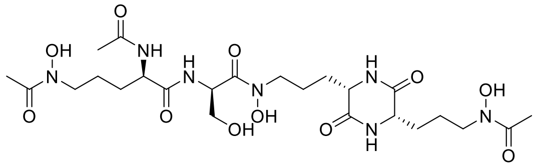

(2)

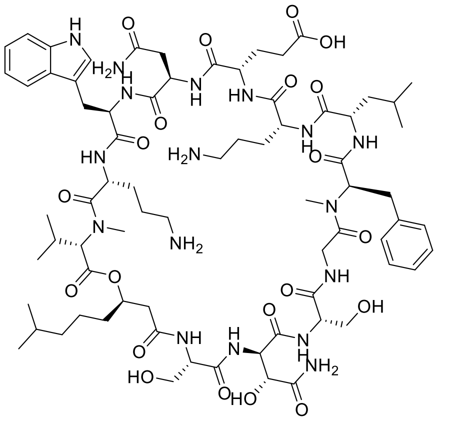

(3)

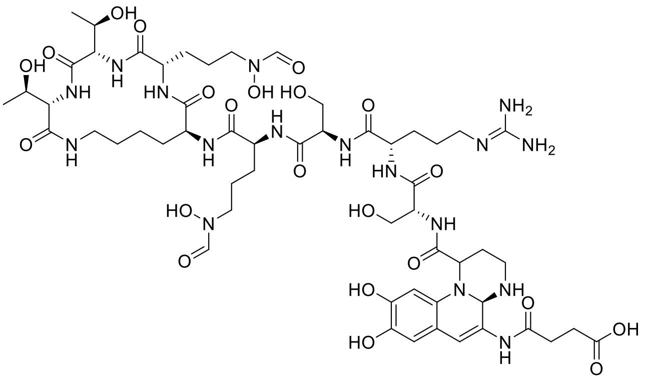

(4)

Figure S5 - For AD domain, 5 OTU sequences were associated with over 85% of identity to BGCs involved in the production of 4 different compounds. Their chemical structures, presented here, were retrieved using ChemDraw v19.0.1.28. (1) – aurantamycin A, (2) – erythrochelin, (3) - P-8294A2 (lotilibcin), (4) – pyoverdine.

References

1. Padeiro, A.; Amaro, E.; Dos Santos, M.M.C.; Araújo, M.F.; Gomes, S.S.; Leppe, M.; Verkulich, S.; Hughes, K.A.; Peter, H.U.; Canário, J. Trace element contamination and availability in the Fildes Peninsula, King George Island, Antarctica. *Environ. Sci. Process. Impacts* **2016**, *18*, 648–657.

2. Kirst, H.A.; Michel, K.H.; Mynderase, J.S.; Chio, E.H.; Yao, R.C.; Nakasukasa, W.M.; Boeck, L.D.; Occlowitz, J.L.; Paschal, J.W.; Deeter, J.B.; et al. Discovery, Isolation, and Structure Elucidation of a Family of Structurally Unique, Fermentation-Derived Tetracyclic Macrolides. In *Synthesis and Chemistry of Agrochemicals III*; 1992; pp. 214–225.

3. Tomita, K.; Oda, N.; Hoshino, Y.; Ohkusa, N.; Chikazawa, H. Fluvirucins A1, A2, B1, B2, B3, B4 and B5, new antibiotics active against influenza a virus. IV. Taxonomy on the producing organisms. *J. Antibiot. (Tokyo).* **1991**, *44*, 940–948.

4. Hu, Y.; Wang, M.; Wu, C.; Tan, Y.; Li, J.; Hao, X.; Duan, Y.; Guan, Y.; Shang, X.; Wang, Y.; et al. Identification and Proposed Relative and Absolute Configurations of Niphimycins C-E from the Marine-Derived Streptomyces sp. IMB7-145 by Genomic Analysis. *J. Nat. Prod.* **2018**, *81*, 178–187.

5. Kim, J.W.; Adachi, H.; Shin-Ya, K.; Hayakawa, Y.; Seto, H. Apoptolidin, a New Apoptosis Inducer in Transformed Cells from Nocardiopsis sp. *J. Antibiot. (Tokyo).* **1997**, *50*, 628–630.

6. Wu, K.; Chung, L.; Revill, W.P.; Katz, L.; Reeves, C.D. The FK520 gene cluster of Streptomyces hygroscopicus var. ascomyceticus (ATCC 14891) contains genes for biosynthesis of unusual polyketide extender units. *Gene* **2000**, *251*, 81–90.

7. Igarashi, Y.; Zhou, T.; Sato, S.; Matsumoto, T.; Yu, L. Akaeolide , a Carbocyclic Polyketide from Marine-Derived Streptomyces. *Org. Lett.* **2013**, *15*, 5678–5681.

8. Robbins, N.; Spitzer, M.; Wang, W.; Waglechner, N.; Patel, D.J.; O'Brien, J.S.; Ejim, L.; Ejim, O.; Tyers, M.; Wright, G.D. Discovery of Ibomycin, a Complex Macrolactone that Exerts Antifungal Activity by Impeding Endocytic Trafficking and Membrane Function. *Cell Chem. Biol.* **2016**, *23*, 1383–1394.

9. Sun, Y.; Xiufen, Z.; Liu, J.; Deng, Z.; Zhang, G.; Kieser, T.; Zhou, X.; Sun, Y.; Bao, K.; Tu, G. 'Streptomyces nanchangensis', a producer of the insecticidal polyether antibiotic nanchangmycin and the antiparasitic macrolide meilingmycin, contains multiple polyketide gene clusters. *Microbiology* **2002**, *148*, 361–371.

10. Kunze, B.; Jansen, R.; Hofle, G.; Reichenbach, H. Ajudazols , New Inhibitors Mitochondrial of the from Electron Transport. *J. Antibiot. (Tokyo).* **2004**, *57*, 151–155.

11. Salituro, G.M.; Zink, D.L.; Dahl, A.; Nielsen, J.; Wu, E.; Huang, L.; Kastner, C.; Dumont, F.J. Meridamycin: A novel nonimmunosuppressive FKBP12 ligand from streptomyces hygroscopicus. *Tetrahedron Lett.* **1995**, *36*, 997–1000.

12. Berger, J.; Rachlin, A.I.; Scott, W.E.; Sternbach, L.H.; Goldberg, M.W. The Isolation of Three New Crystalline Antibiotics from Streptomyces. *J. Am. Chem. Soc.* **1951**, *73*, 5295–5298.

13. Park, H.R.; Furihata, K.; Hayakawa, Y.; Shin-Ya, K. Versipelostatin, a novel GRP78/Bip molecular chaperone down-regulator of microbial origin. *Tetrahedron Lett.* **2002**, *43*, 6941–6945.

14. Ikemoto, T.; Katayama, T.; Shiraishi, A.; Haneishi, T. Aculeximycin, a new antibiotic from Streptosporangium albidum. II. isolation, physicochemical and biological properties. *J. Antibiot. (Tokyo).* **1983**, *36*, 1097–1100.

15. Helfrich, E.J.N.; Vogel, C.M.; Ueoka, R.; Schäfer, M.; Ryffel, F.; Müller, D.B.; Probst, S.; Kreuzer, M.; Piel, J.; Vorholt, J.A. Bipartite interactions, antibiotic production and biosynthetic potential of the Arabidopsis leaf microbiome. *Nat. Microbiol.* **2018**, *3*, 909–919.

16. Cai, P.; Kong, F.; Ruppen, M.E.; Glasier, G.; Carter, G.T. Hygrocins A and B, Naphthoquinone Macrolides from Streptomyces hygroscopicus. *J. Nat. Prod.* **2005**, *68*, 1736–1742.

17. Zhou, T.; Komaki, H.; Ichikawa, N.; Hosoyama, A.; Sato, S.; Igarashi, Y. Biosynthesis of akaeolide and lorneic acids and annotation of type i polyketide synthase gene clusters in the genome of Streptomyces sp. NPS554. *Mar. Drugs* **2015**, *13*, 581–596.

18. Banskota, A.H.; McAlpine, J.B.; Sørensen, D.; Aouidate, M.; Piraee, M.; Alarco, A.M.; Omura, S.; Shiomi, K.; Farnet, C.M.; Zazopoulos, E. Isolation and identification of three new 5-alkenyl-3,3(2H)-furanones from two Streptomyces species using a genomic screening approach. *J. Antibiot. (Tokyo).* **2006**, *59*, 168–176.

19. Viehrig, K.; Surup, F.; Volz, C.; Herrmann, J.; Abou Fayad, A.; Adam, S.; Köhnke, J.; Trauner, D.; Müller, R. Structure and Biosynthesis of Crocagins: Polycyclic Posttranslationally Modified Ribosomal Peptides from Chondromyces crocatus. *Angew. Chemie - Int. Ed.* **2017**, *56*, 7407–7410.

20. Gerwick, W.H.; Proteau, P.J.; Nagle, D.G.; Hamel, E.; Blokhin, A.; Slate, D.L. Structure of Curacin A, a Novel Antimitotic, Antiproliferative and Brine Shrimp Toxic Natural Product from the Marine Cyanobacterium Lyngbya majuscula. *J. Org. Chem.* **1994**, *59*, 1243–1245.

21. Shichijo, Y.; Migita, A.; Oguri, H.; Watanabe, M.; Tokiwano, T.; Watanabe, K.; Oikawa, H. Epoxide Hydrolase Lsd19 for Polyether Formation in the Biosynthesis of Lasalocid A: Direct Experimental Evidence on Polyene-Polyepoxide Hypothesis in Polyether Biosynthesis. *J. Am. Chem. Soc.* **2008**, *130*, 12230–12231.

22. Li, P.; Li, J.; Arikan, F.; Ahlbrecht, W.; Dieckmann, M.; Menche, D. Total synthesis of etnangien. *J. Am. Chem. Soc.* **2009**, *131*, 11678–11679.

23. Hardner, R.L.; Hidy, P.H.; Corum, C.J.; Jones, K.L. Nigericin a new crystalline antibiotic from an unidentified Streptomyces. *Antibiot Chemother (Northfield)* **1951**, *1*, 594–6.

24. He, Y.; Cox, R.J. The molecular steps of citrinin biosynthesis in fungi. *Chem. Sci.* **2016**, *7*, 2119–2127.

25. Ohlendorf, B.; Leyers, S.; Krick, A.; Kehraus, S.; Wiese, M.; König, G.M. Phenylnannolones A-C: biosynthesis of new secondary metabolites from the myxobacterium Nannocystis exedens. *Chembiochem* **2008**, *9*, 2997–3003.

26. Rateb, M.E.; Houssen, W.E.; Arnold, M.; Abdelrahman, M.H.; Deng, H.; Harrison, W.T.A.; Okoro, C.K.; Asenjo, J.A.; Andrews, B.A.; Ferguson, G.; et al. Chaxamycins A - D, bioactive ansamycins from a hyper-arid desert streptomyces sp. *J. Nat. Prod.* **2011**, *74*, 1491–1499.

27. Igarashi, M.; Tsuchida, T.; Kinoshita, N.; Kamijima, M.; Sawa, R.; Sawa, T.; Naganawa, H.; Hamada, M.; Takeuchi, T.; Yamazaki, K.; et al. Cremimycin, a novel 19-membered macrocyclic lactam antibiotic, from Streptomyces sp. *J. Antibiot. (Tokyo).* **1998**, *51*, 123–129.

28. Yuan, Y.; Men, H.; Lee, C. Total synthesis of kendomycin: A macro-C-glycosidation approach. *J. Am. Chem. Soc.* **2004**, *126*, 14720–14721.

29. Theodore, C.M.; Stamps, B.W.; King, J.B.; Price, L.S.L.; Powell, D.R.; Stevenson, B.S.; Cichewicz, R.H. Genomic and metabolomic insights into the natural product biosynthetic diversity of a feral-hog-associated Brevibacillus laterosporus strain. *PLoS One* **2014**, *9*, 3–12.

30. Kamigiri, K.; Suzuki, Y.; Shibazaki, M.; Morioka, M.; Suzuki, K.I.; Tokunaga, T.; Setiawan, B.; Rantiatmodjo, R.M. Kalimantacins A, B and C, novel antibiotics from Alcaligenes sp. YL-02632S I. Taxonomy, fermentation, isolation and biological properties. *J. Antibiot. (Tokyo).* **1996**, *49*, 136–139.

31. Bui, H.T.N.; Jansen, R.; Pham, H.T.L.; Mundt, S. Carbamidocyclophanes A-E, chlorinated paracyclophanes with cytotoxic and antibiotic activity from the Vietnamese cyanobacterium Nostoc sp. *J. Nat. Prod.* **2007**, *70*, 499–503.

32. Yang, J.; Zhu, X.; Cao, M.; Wang, C.; Zhang, C.; Lu, Z.; Lu, F. Genomics-Inspired Discovery of Three Antibacterial Active Metabolites, Aurantinins B, C, and D from

Compost-Associated Bacillus subtilis fmb60. *J. Agric. Food Chem.* **2016**, *64*, 8811–8820.

33. Mareš, J.; Hájek, J.; Urajová, P.; Kopecký, J.; Hrouzek, P. A hybrid non-ribosomal peptide/polyketide synthetase containing fatty-acyl ligase (FAAL) synthesizes the β-amino fatty acid lipopeptides puwainaphycins in the Cyanobacterium Cylindrospermum alatosporum. *PLoS One* **2014**, *9*, e111904.

34. Raju, R.; Piggott, A.M.; Conte, M.M.; Capon, R.J. Heronamides A - C, new polyketide macrolactams from an Australian marine-derived Streptomyces sp. A biosynthetic case for synchronized tandem electrocyclization. *Org. Biomol. Chem.* **2010**, *8*, 4682–4689.

35. Jahns, C.; Hoffmann, T.; Müller, S.; Gerth, K.; Washausen, P.; Höfle, G.; Reichenbach, H.; Kalesse, M.; Müller, R. Pellasoren: Structure elucidation, biosynthesis, and total synthesis of a cytotoxic secondary metabolite from Sorangium cellulosum. *Angew. Chemie - Int. Ed.* **2012**, *51*, 5239–5243.

36. Nozoe, S.; Kikuchi, K.; Ishii, N.; Ohta, T. Synthesis of neocarzilin A: An absolute streochemistry. *Tetrahedron Lett.* **1992**, *33*, 7551–7552.

37. Irschik, H.; Gerth, K.; Reichenbach, H.; Jansen, R.; Hofle, G. The Sorangicins, Novel And Powerful Inhibitors Of Eubacterial Rna Polymerase Isolated From Myxobacteria. *J. Antibiot. (Tokyo).* **1987**, *40*, 7–13.

38. Newbold, C.J.; Wallace, R.J.; Watt, N.D.; Richardson, A.J. Effect of the novel ionophore tetronasin (ICI 139603) on ruminal microorganisms. *Appl. Environ. Microbiol.* **1988**, *54*, 544–547.

39. Wu, X.C.; Qian, C.D.; Fang, H.H.; Wen, Y.P.; Zhou, J.Y.; Zhan, Z.J.; Ding, R.; Li, O.; Gao, H. Paenimacrolidin, a novel macrolide antibiotic from Paenibacillus sp. F6-B70 active against methicillin-resistant Staphylococcus aureus. *Microb. Biotechnol.* **2011**, *4*, 491–502.

40. Oh, D.C.; Poulsen, M.; Currie, C.R.; Clardy, J. Sceliphrolactam, a polyene macrocyclic lactam from a wasp-associated Streptomyces sp. *Org. Lett.* **2011**, *13*, 752–755.

41. Jurkiewicz, E.; Jansen, R.; Kunze, B.; Trowitzsch-Kienast, W.; Forche, E.; Reichenbach, H.; Hofle, G.; Hunsmann, G. Three new potent HIV-1 inhibitors from myxobacteria. *Antivir. Chem. Chemother.* **1992**, *3*, 189–193.

42. Thierbach, G.; Reichenbach, H. Myxothiazol, a new antibiotic interfering with respiration. *Antimicrob. Agents Chemother.* **1981**, *19*, 504–507.

43. Kalan, L.; Gessner, A.; Thaker, M.N.; Waglechner, N.; Zhu, X.; Szawiola, A.; Bechthold, A.; Wright, G.D.; Zechel, D.L. A cryptic polyene biosynthetic gene cluster in streptomyces calvus is expressed upon complementation with a functional bldA gene. *Chem. Biol.* **2013**, *20*, 1214–1224.

44. Gao, J.M.; Yang, S.X.; Qin, J.C. Azaphilones: Chemistry and biology. *Chem. Rev.* **2013**, *113*, 4755–4811.

45. Sheehan, J.; Murphy, C.D.; Caffrey, P. New insights into polyene macrolide biosynthesis in Couchioplanes caeruleus. *Mol. Biosyst.* **2017**, *13*, 866–873.

46. Kunze, B.; Kemmer, T.; Höfle, G.; Reichenbach, H. Stigmatellin, a new antibiotic from stigmatella aurantiaca (myxobacterales): I. production, physico-chemical and biological properties. *J. Antibiot. (Tokyo).* **1984**, *37*, 454–461.

47. May, D.S.; Chen, W.-L.; Lantvit, D.D.; Zhang, X.; Krunic, A.; Burdette, J.E.; Eustaquio, A.; Orjala, and J. Merocyclophanes C and D from the Cultured Freshwater Cyanobacterium

Nostoc sp. (UIC 10110) Daniel. *J Nat Prod.* **2017**, *80*, 1073–1080.

48. Gräfe, U.; Schlegel, R.; Ritzau, M.; Ihn, W.; Dornberger, K.; Stengel, C.; Fleck, W.F.; Gutsche, W.; Härtl, A.; Paulus, E.F. Aurantimycins, new depsipeptide antibiotics from Streptomyces aurantiacus IMET 43917. Production, isolation, structure elucidation, and biological activity. *J. Antibiot. (Tokyo).* **1995**, *48*, 119–25.

49. Robbel, L.; Knappe, T.A.; Linne, U.; Xie, X.; Marahiel, M.A. Erythrochelin-a hydroxamate-type siderophore predicted from the genome of Saccharopolyspora erythraea. *FEBS J.* **2010**, *277*, 663–676.

50. Kato, A.; Nakaya, S.; Ohashi, Y.; Hirata, H.; Fujii, K.; Harada, K. WAP-8294A2, a novel anti-MRSA antibiotic produced by Lysobacter sp. *J. Am. Chem. Soc.* **1997**, *119*, 6680–6681.

51. Stintzi, A.; Pierre, C.; Hohnadel, D.; Meyer, J.; Dean, C.; Poole, K.; Kourambas, S.; Krishnapillai, V. Novel pyoverdine biosynthesis gene(s) of Pseudomonas aeruginosa PA0. *Microbiology* **1996**, *142*, 1181–1190.

52. Umezawa, K.; Nakazawa, K.; Uemura, T.; Ikeda, Y.; Kondo, S.; Naganawa, H.; Kinoshita, N.; Hashizume, H.; Hamada, M.; Takeuchi, T.; et al. Polyoxypeptin isolated from Streptomyces: A bioactive cyclic depsipeptide containing the novel amino acid 3-hydroxy-3-methylproline. *Tetrahedron Lett.* **1998**, *39*, 1389–1392.

53. Laycock, M. V.; Hildebrand, P.D.; Thibault, P.; Walter, J.A.; Wright, J.L.C. Viscosin, a potent peptidolipid biosurfactant and phytopathogenic mediator produced by a pectolytic strain of Pseudomonas fluorescens. *J. Agric. Food Chem.* **2005**, *39*, 483–489.

54. Weckesser, J.; Martin, C.; Jakobi, C. Cyanopeptolins, depsipeptides from cyanobacteria. *Syst. Appl. Microbiol.* **1996**, *19*, 133–138.

55. McBrien, K.D.; Berry, R.L.; Lowe, S.E.; Neddermann, K.M.; Bursuker, I.; Steven, S.H.; Klohr, S.E.; Leet, J.E. Rakicidins, New Cytotoxic Lipopeptides from Micromonospora sp. Fermentation, Isolation and Characterization. *J. Antibiot. (Tokyo).* **1995**, *48*, 1446–1452.

56. Hamamoto, H.; Urai, M.; Ishii, K.; Yasukawa, J.; Paudel, A.; Murai, M.; Kaji, T.; Kuranaga, T.; Hamase, K.; Katsu, T.; et al. Lysocin e is a new antibiotic that targets menaquinone in the bacterial membrane. *Nat. Chem. Biol.* **2015**, *11*, 127–133.

57. Herfindal, L.; Myhren, L.; Kleppe, R.; Krakstad, C.; Selheim, F.; Jokela, J.; Sivonen, K.; Døskeland, S.O. Nostocyclopeptide-M1: A potent, nontoxic inhibitor of the hepatocyte drug transporters OATP1B3 and OATP1B1. *Mol. Pharm.* **2011**, *8*, 360–367.

58. Wang, X.; Zhou, H.; Chen, H.; Jing, X.; Zheng, W.; Li, R.; Sun, T.; Liu, J.; Fu, J.; Huo, L.; et al. Discovery of recombinases enables genome mining of cryptic biosynthetic gene clusters in Burkholderiales species. *Proc. Natl. Acad. Sci.* **2018**, *115*, E4255–E4263.
